# Supplementary material for: Phasic Dopamine Changes and Hebbian Mechanisms during Probabilistic Reversal Learning in Striatal Circuits: A Computational Study
Source: Int J Mol Sci. 2022 Mar 22;23(7):3452. doi: 10.3390/ijms23073452 (PMC8998230; doi:10.3390/ijms23073452)
Supplement: Supplementary file 1 [file ijms-23-03452-s001.zip › ijms-1614705-supplementary/Supplementary Material/Supplementary Material III_Synapses_ijms.pdf]

Supplementary Materials SIII: Synapses

PHASIC DOPAMINE CHANGES AND HEBBIAN MECHANISMS DURING PROBABILISTIC REVERSAL LEARNING IN STRIATAL CIRCUITS: A COMPUTATIONAL STUDY

Authors

Miriam Schirru[1], Florence Véronneau-Veilleux[2], Fahima Nekka[2 3 4], Mauro Ursino[1]

Affiliations:

1) Department of Electrical, Electronic and Information Engineering Guglielmo Marconi, University of Bologna, Campus of Cesena, I 47521 Cesena, Italy

2) Faculté de Pharmacie, Université de Montréal, Montréal, Québec H3T 1J4, Canada

3) Centre de recherches mathématiques, Université de Montréal, Montréal, Québec H3T 1J4, Canada

4) Centre for Applied Mathematics in Bioscience and Medicine (CAMBAM), McGill University, Montréal, Québec H3G 1Y6, Canada

Corresponding author - Mauro Ursino: mauro.ursino@unibo.it

Figure S3 – Temporal pattern of the synapses from the motor cortex to the Go (W^{GC}), from the Sensory cortex to the Go (W^{GS}), from the motor cortex to the NoGo (W^{NC}), and from the sensory cortex to the NoGo (W^{NS}) during a two-choice basal training, performed with the new equations for dopamine phasic changes (Equations (1)-(3) in the main text). Figures refer to a single subject, representative of the ten subjects simulated during the work.

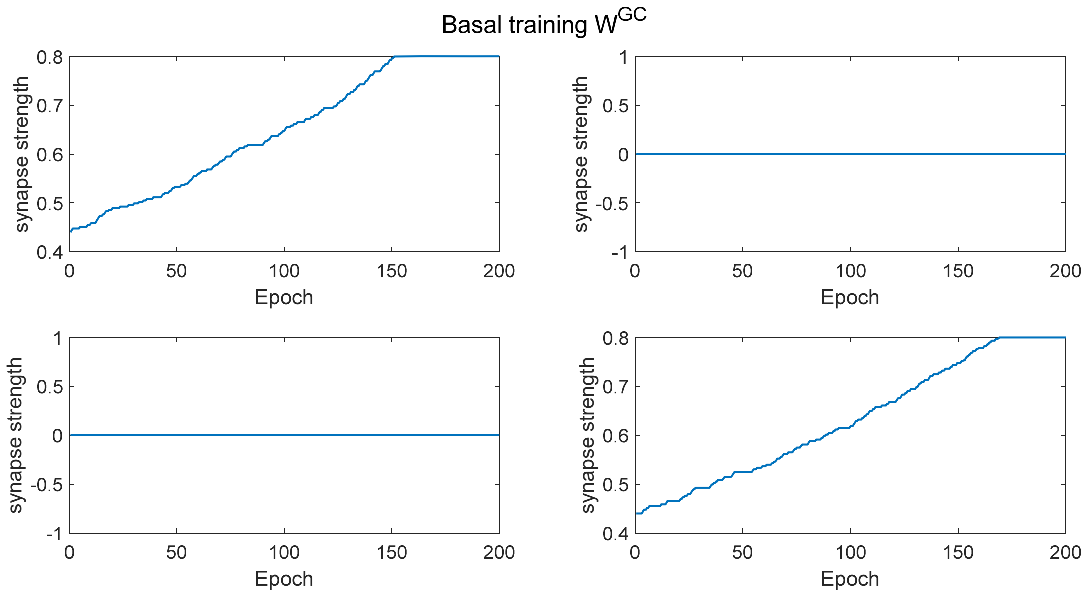

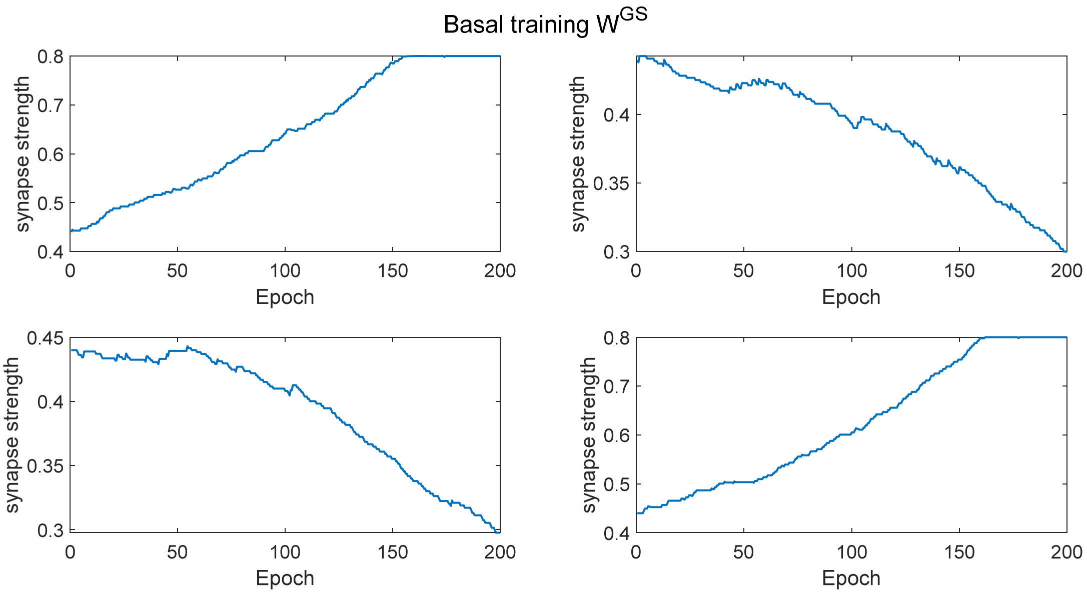

Basal training W^{NC}

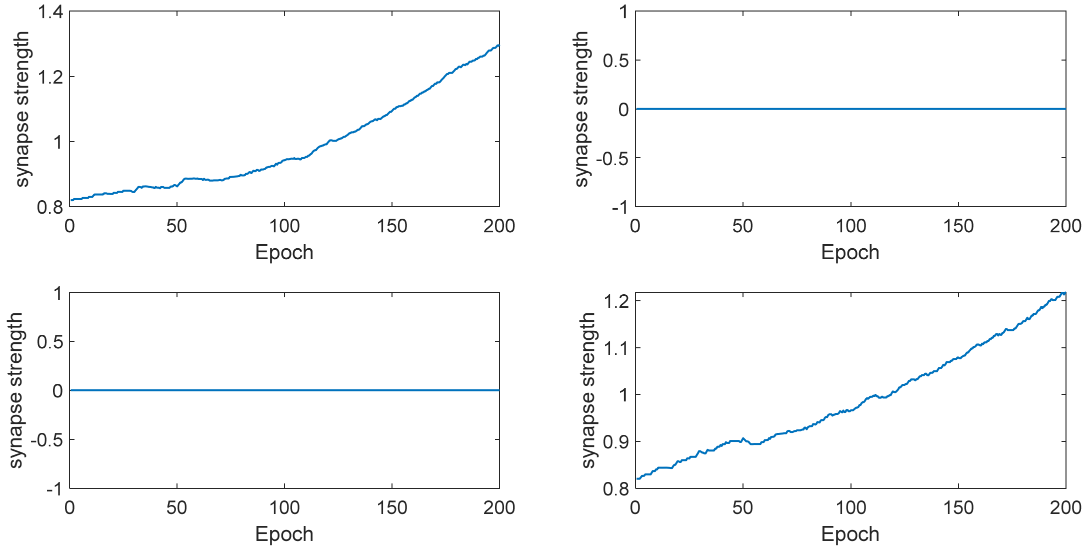

Basal training W^{NS}

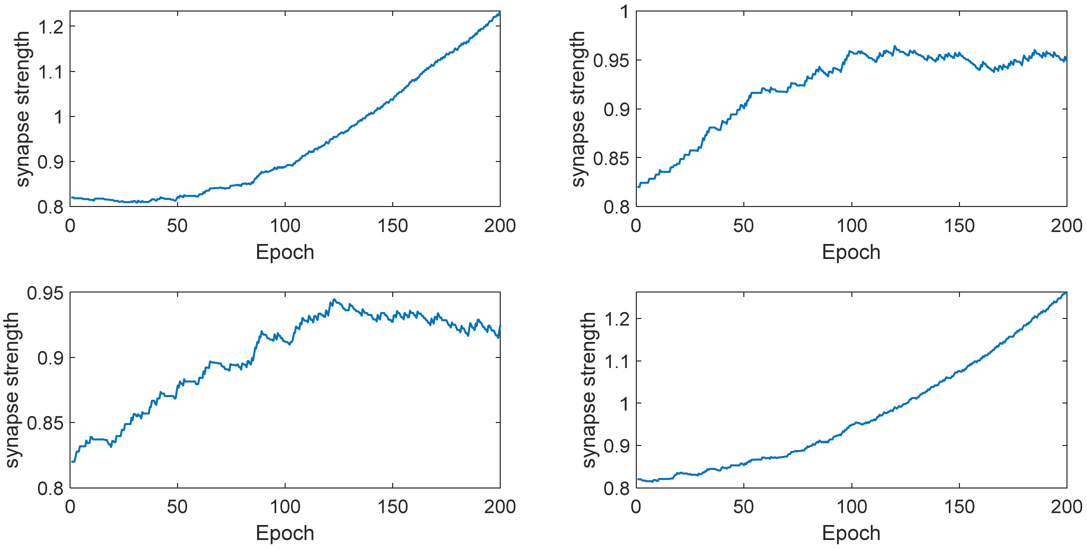

Figure S4 – Temporal pattern of the synapses from the motor cortex to the Go (W^{GC}), from the Sensory cortex to the Go (W^{GS}), from the motor cortex to the NoGo (W^{NC}), and from the sensory cortex to the NoGo (W^{NS}) during a two-choice reversal training, performed after 75 previous epochs of basal training, with the new equations for dopamine phasic changes (Equations (1)-(3) in the main text). Figures refer to a single subject, the same as in Figure S3, representative of the ten subjects simulated during the work.

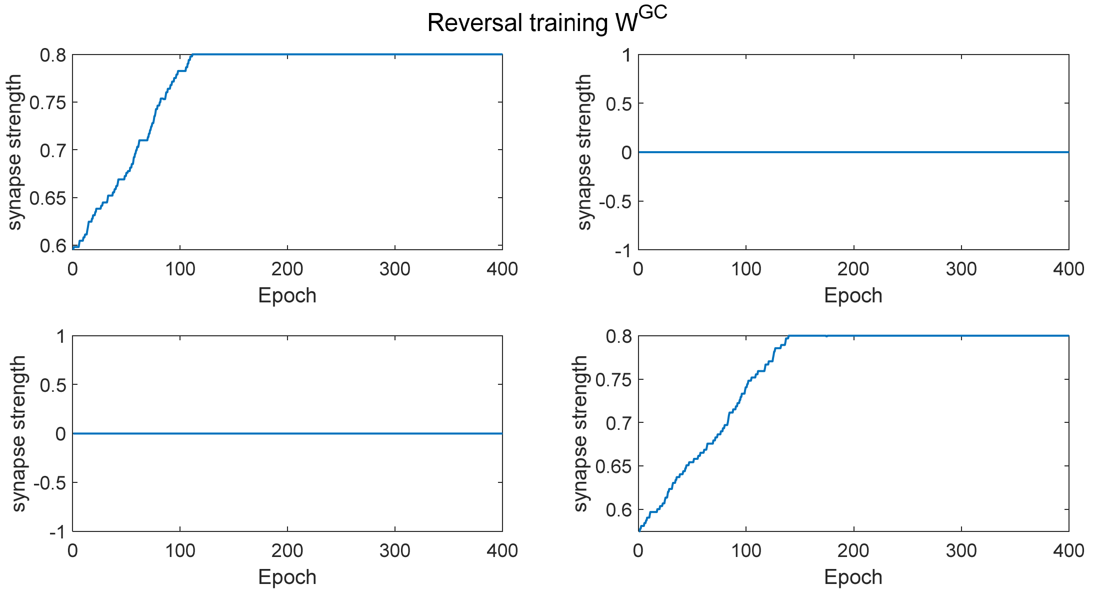

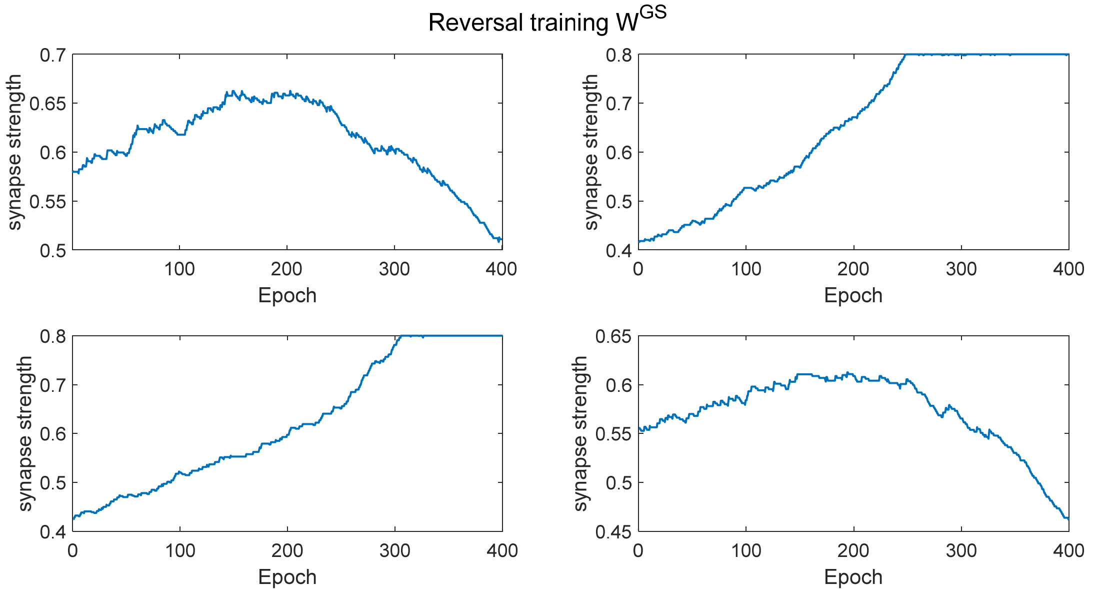

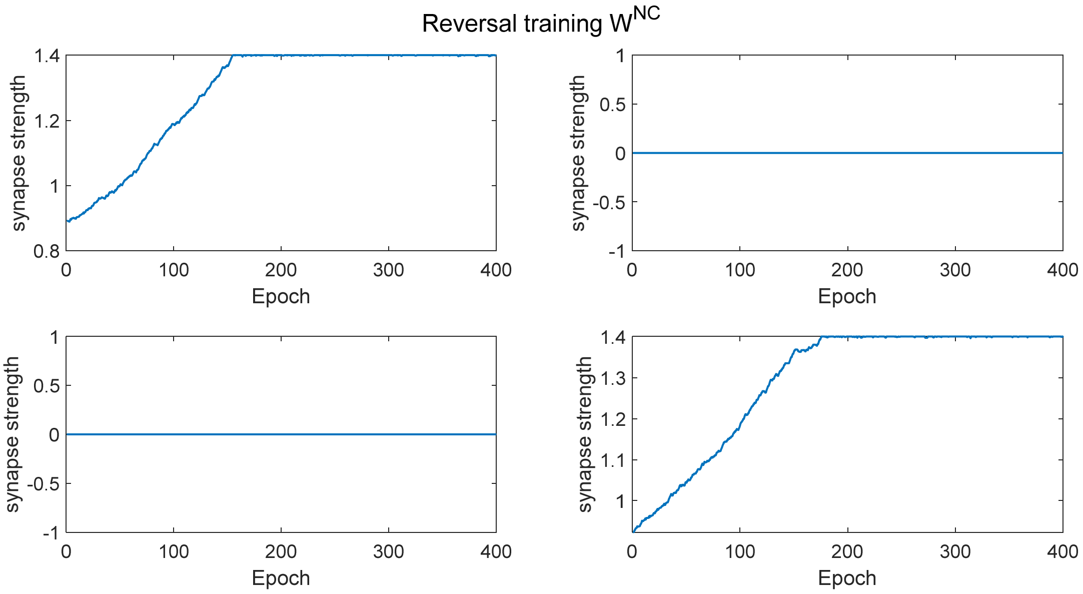

Reversal training W^NC
synapse strength
Epoch

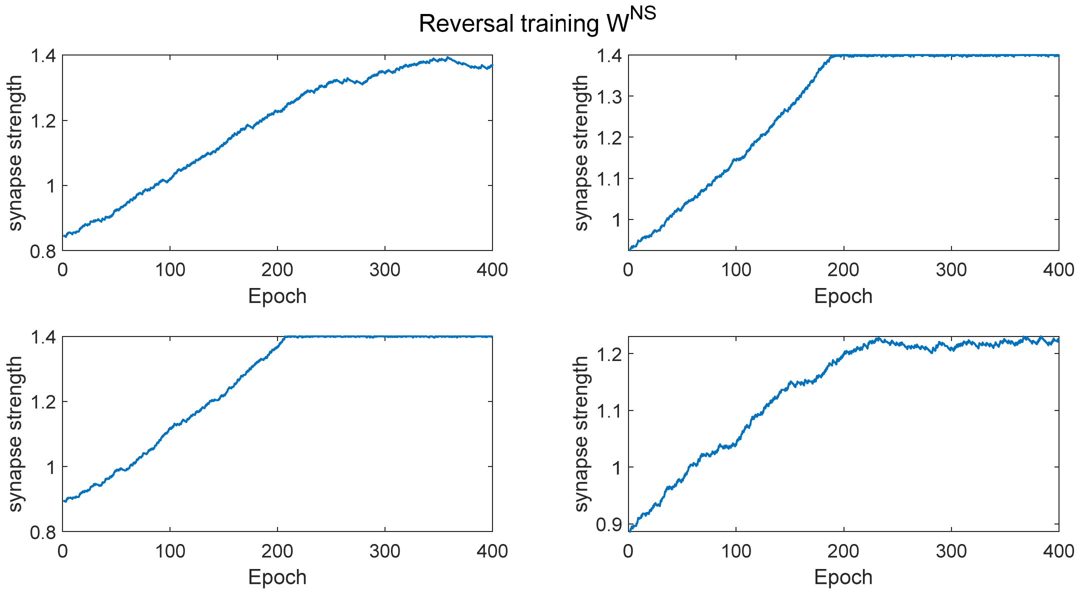

Reversal training W^NS
synapse strength
Epoch
