# Supplementary material for: Phasic Dopamine Changes and Hebbian Mechanisms during Probabilistic Reversal Learning in Striatal Circuits: A Computational Study
Source: Int J Mol Sci. 2022 Mar 22;23(7):3452. doi: 10.3390/ijms23073452 (PMC8998230; doi:10.3390/ijms23073452)
Supplement: Supplementary file 1 [file ijms-23-03452-s001.zip › ijms-1614705-supplementary/Supplementary Material/Supplementary Material II_Rules_ijms.pdf]

Supplementary Materials SII: Hebb rules

PHASIC DOPAMINE CHANGES AND HEBBIAN MECHANISMS DURING PROBABILISTIC REVERSAL LEARNING IN STRIATAL CIRCUITS: A COMPUTATIONAL STUDY

Authors

Miriam Schirru[1], Florence Véronneau-Veilleux[2], Fahima Nekka[2 3 4], Mauro Ursino[1]

Affiliations:

1) Department of Electrical, Electronic and Information Engineering Guglielmo Marconi, University of Bologna, Campus of Cesena, I 47521 Cesena, Italy

2) Faculté de Pharmacie, Université de Montréal, Montréal, Québec H3T 1J4, Canada

3) Centre de recherches mathématiques, Université de Montréal, Montréal, Québec H3T 1J4, Canada

4) Centre for Applied Mathematics in Bioscience and Medicine (CAMBAM), McGill University, Montréal, Québec H3G 1Y6, Canada

Corresponding author - Mauro Ursino: mauro.ursino@unibo.it

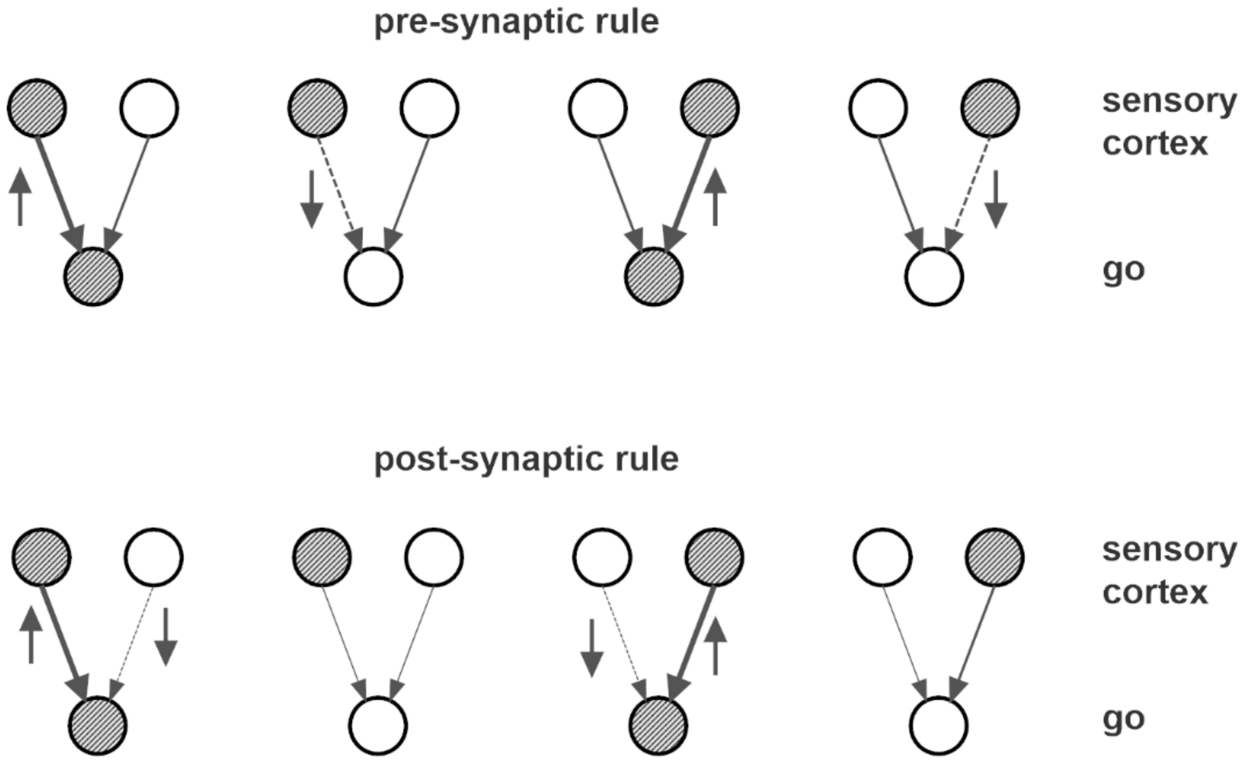

Figure S1 - Comparison between the pre-synaptic and post-synaptic rules, considering the synapses from the *sensory cortex* into a *Go* neuron (hence, just a single action channel is shown). The examples refer to an input stimulus with two components. Circles describe neural units (pre-synaptic in the sensory cortex, post-synaptic as to the Go neuron). Filled circles signify that the neuron is active (above the threshold for the Hebb rule) whereas white circles refer to non-active neurons (below threshold). All the four possible conditions occurring in practice are presented. Thick lines describe synapses that are reinforced (arrow up), and dashed lines synapses which are weakened (arrow down). The other synapses do not change their values as a consequence of the function "positive part" used for the pre-synaptic neurons (upper row) or for the post-synaptic neuron (bottom row).

It is worth noting that a Go neuron can be above threshold only if it is rewarded, but can be below threshold either if it is punished or if the action channel has not been selected. Hence, in a naïve network, a non-active Go neuron is more frequent than an active one.

In the case of the pre-synaptic rule, a synapse previously reinforced can be subsequently weakened, if the Go neuron is not active (a condition frequently occurring in a naïve network as explained above, resulting in poor synapse learning). Conversely, in the post-synaptic rule, the synapses are modified only when the neuron is rewarded (hence above threshold), resulting in the simultaneous reinforcement of the necessary synapses and weakening of the unnecessary ones.

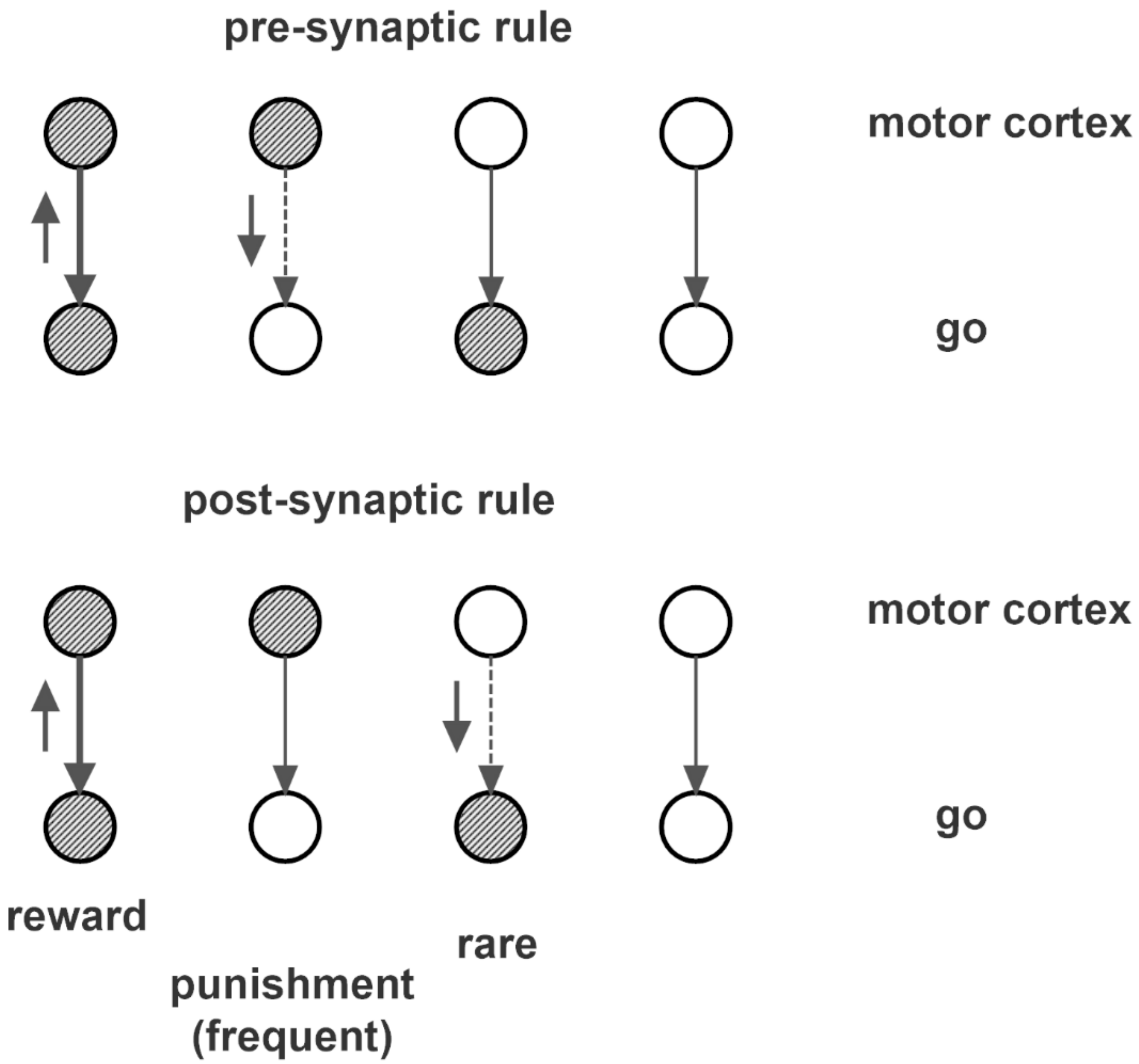

Figure S2 - Comparison between the pre-synaptic and post-synaptic rules, considering the synapses from the *motor cortex* into a *Go* neuron (hence, just a single action channel is shown). Since the action channels are segregated, there is only one synapse from the corresponding motor neuron to the Go neuron. Circles describe neural units (pre-synaptic in the motor cortex, post-synaptic as to the Go neuron). Filled circles signify that the neuron is active (above the threshold for the Hebb rule) whereas white circles refer to non-active neurons (below threshold). All the four possible conditions occurring in practice are presented. Thick lines describe synapses that are reinforced (arrow up), and dashed lines synapses which are weakened (arrow down). The other synapses do not change their values as a consequence of the function "positive part" used for the pre-synaptic neurons (upper row) or for the post-synaptic neuron (bottom row).

The condition in which a motor neuron is silent while the Go neuron is active rarely occurs in a naïve network, since the Go neuron needs the input from the cortex to overcome the threshold. Conversely, the condition in which a motor neuron is active but the Go neuron is under threshold can occur after a punishment, a condition quite frequent in the four-channel case for a naïve network, resulting in synapse weakening.
