# Supplementary material for: Phasic Dopamine Changes and Hebbian Mechanisms during Probabilistic Reversal Learning in Striatal Circuits: A Computational Study
Source: Int J Mol Sci. 2022 Mar 22;23(7):3452. doi: 10.3390/ijms23073452 (PMC8998230; doi:10.3390/ijms23073452)

Supplementary Materials SIV: role of parameter m

PHASIC DOPAMINE CHANGES AND HEBBIAN MECHANISMS DURING PROBABILISTIC REVERSAL LEARNING IN STRIATAL CIRCUITS: A COMPUTATIONAL STUDY

Authors

Miriam Schirru[1], Florence Véronneau-Veilleux[2], Fahima Nekka[2 3 4], Mauro Ursino[1]

Affiliations:

1) Department of Electrical, Electronic and Information Engineering Guglielmo Marconi, University of Bologna, Campus of Cesena, I 47521 Cesena, Italy

2) Faculté de Pharmacie, Université de Montréal, Montréal, Québec H3T 1J4, Canada

3) Centre de recherches mathématiques, Université de Montréal, Montréal, Québec H3T 1J4, Canada

4) Centre for Applied Mathematics in Bioscience and Medicine (CAMBAM), McGill University, Montréal, Québec H3G 1Y6, Canada

Corresponding author - Mauro Ursino: mauro.ursino@unibo.it

Figure S5 – Role of parameter *m* - Results of the tests performed after basal training and after a reversal learning, obtained by using the Equations (2) and (3) to automatically compute the phasic changes in the dopaminergic input as a function of the expected reward. The panels refer to a 2-choice task (100 trials per each test) and should be compared with the curves in Figure 6. These figures differ from those in Figure 11 (upper panels) since have been performed with a value $m = 1$ in Equations (2)-(3), i.e., assuming a subject with smaller phasic changes in dopamine as a function of the expected reward. The reversal learning does not work properly after 75 previous epochs, due to insufficient punishment strength.

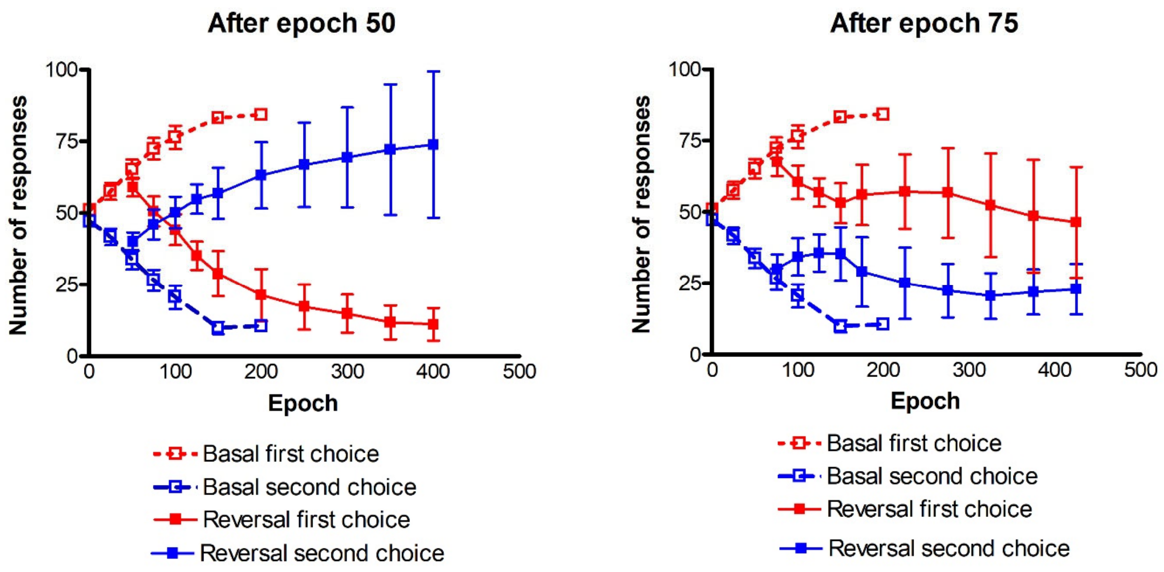

Supplement: Supplementary file 1 [file ijms-23-03452-s001.zip › ijms-1614705-supplementary/Supplementary Material/Supplementary Material IV_Role of m_ijms.pdf]
