# Supplementary material for: Phasic Dopamine Changes and Hebbian Mechanisms during Probabilistic Reversal Learning in Striatal Circuits: A Computational Study
Source: Int J Mol Sci. 2022 Mar 22;23(7):3452. doi: 10.3390/ijms23073452 (PMC8998230; doi:10.3390/ijms23073452)
Supplement: Supplementary file 1 [file ijms-23-03452-s001.zip › ijms-1614705-supplementary/Supplementary Material/Supplementary Material I_Model_ijms.pdf]

Supplementary Materials SI: Model

PHASIC DOPAMINE CHANGES AND HEBBIAN MECHANISMS DURING PROBABILISTIC REVERSAL LEARNING IN STRIATAL CIRCUITS: A COMPUTATIONAL STUDY

Authors

Miriam Schirru[1], Florence Véronneau-Veilleux[2], Fahima Nekka[2 3 4], Mauro Ursino[1]

Affiliations:

1) Department of Electrical, Electronic and Information Engineering Guglielmo Marconi, University of Bologna, Campus of Cesena, I 47521 Cesena, Italy

2) Faculté de Pharmacie, Université de Montréal, Montréal, Québec H3T 1J4, Canada

3) Centre de recherches mathématiques, Université de Montréal, Montréal, Québec H3T 1J4, Canada

4) Centre for Applied Mathematics in Bioscience and Medicine (CAMBAM), McGill University, Montréal, Québec H3G 1Y6, Canada

Corresponding author - Mauro Ursino: mauro.ursino@unibo.it

Individual neuron dynamics

Let i be a post-synaptic neuron, which receives synapses w_{ij} from pre-synaptic neurons j, whose activity is y_j. Additional inputs coming from external sources are summarized in a single term I_i.

Every input to the neuron i converges in a variable x_i. Assuming N pre-synaptic neurons projecting to the post-synaptic neuron i, we can write:

$$x_i = \sum_{j=1}^{N} w_{ij} y_j + I_i \quad (1)$$

The input x_i is then transformed into a post-synaptic variable u_i using a first order differential equation with time constant τ, in order to mimic the cell membrane integrative process

$$\tau \frac{du_i}{dt} = -u_i + x_i \quad (2)$$

As last step, a sigmoidal function ς computes the activity of the neuron i, y_i, from the output of the previous differential equation u_i

$$y_i = \varsigma(u_i) \quad (3)$$

implemented as

$$y_i = \frac{1}{1+e^{-a(u_i-u_0)}} \quad (4)$$

being a and u_0 parameters which set the central slope and the central position of the sigmoid.

Network connectivity

For the majority of the layers (S, C, Go, NoGo, Gpe, Gpi), synapses are represented with the symbol W and two superscripts plus two subscripts. The two subscripts (i, j with i, j = 1, 2, …, N) specify the position of the post-synaptic and pre-synaptic neurons, respectively. N denotes the number of action channels, i.e., the number of possible conflicting actions to be chosen. As in Baston *et al.* (2016) we use $N = 2$, to represent the two possible actions. Superscripts specify the target layer (where the post-synaptic neuron i is located) and the donor layer (where the pre-synaptic neuron j is located).

The STN and the cholinergic interneurons ChI are modeled as single units and therefore they do not need subscripts.

The acronyms are S: sensory cortex; C: motor cortex; T: thalamus; G: Go; N: NoGo; I: Gpi; E: Gpe; H: cholinergic interneurons ChI; STN: sub-thalamic nucleus. L indicates the lateral inhibition within the cortex.

Cortex - We can write for $i = 1, \ldots, N$:

$$\tau_L \frac{du_i^L}{dt} = -u_i^L + \sum_{\substack{j=1 \\ i \neq j}}^{N} l_{ij} y_j^C \tag{5}$$

$$\tau \frac{du_i^C}{dt} = -u_i^C + \sum_{j=1}^{N} w_{ij}^{CS} s_j + u_i^L + w_{ii}^{CT} y_i^T + n_i \tag{6}$$

$$y_i^C = \varsigma(u_i^C) \tag{7}$$

where y_i^C is the activity of a neuron of the cortex C. Every neuron of C receives excitatory inputs from the whole stimulus vector S (each individual component is named s_j), an excitatory projection y_i^T from the corresponding neuron in the thalamus and an additional input u_i^L, representing lateral inhibition from the other neurons in the cortex. A different time constant τ_L characterizes the latter. If the neuron of the thalamus is active, the neuron of the cortex receives the positive feedback and the corresponding action could be gated. n_i is a Gaussian with noise with zero mean value and standard deviation, σ_i , introduced during training to mimic exploration (which is an essential aspect of all learning processes).

Go part of the striatum - We can write for $i = 1, \ldots, N$:

$$\tau \frac{du_i^G}{dt} = -u_i^G + \sum_{j=1}^{N} w_{ij}^{GS} s_j + w_{ii}^{GC} y_i^C + \alpha \cdot D \cdot \left(y_i^G - \vartheta^G\right) + w^{GH} y^H \tag{8}$$

$$y_i^G = \varsigma(u_i^G) \tag{9}$$

where y_i^G is the activity of a neuron of the Go part of the striatum. Every neuron of the Go receives excitatory input from the whole stimulus vector S and an excitatory projection from the corresponding neuron of the cortex C. The direct pathway starts here. Furthermore, activity of each neuron in the

Go is modulated by dopamine (whose effect id denoted as *D*) and by the cholinergic interneuron activity (y^H).

Dopamine is excitatory ($\alpha > 0$) if the Go activity is above threshold (ϑ^G), inhibitory otherwise, thus realizing the contrast enhancement effect (Nicola *et al.*, 2000). The cholinergic interneurons are always inhibitory ($w^{GH} < 0$) to the Go.

NoGo part of the striatum - We have for $i = 1, \dots, N$:

$$\tau \frac{du_i^N}{dt} = -u_i^N + \sum_{j=1}^{N} w_{ij}^{NS} s_j + w_{ii}^{NC} y_i^C + \beta \cdot D + w^{NH} y^H \tag{10}$$

$$y_i^N = \varsigma(u_i^N) \tag{11}$$

where y_i^N is the activity of a neuron of the NoGo part of the striatum. Every neuron of the NoGo receives excitatory input from the whole stimulus S and excitatory projection from the corresponding neuron in the cortex C. The indirect pathway starts here. Furthermore, activity of each neuron in the NoGo is modulated by dopamine and by the cholinergic interneuron activity (y^H).

Dopamine provides inhibition ($\beta < 0$) to all the NoGo neurons, while the cholinergic interneuron provides excitation ($w^{NH} > 0$). Hence, dopamine and acetylcholine exert specular tonic and phasic effects on NoGo activity with respect to the Go case.

Globus pallidus pars externa - Equations are for $i = 1, \dots, N$:

$$\tau \frac{du_i^E}{dt} = -u_i^E + w_{ii}^{EN} y_i^N + w^{ESTN} y^{STN} + I^E \tag{12}$$

$$y_i^E = \varsigma(u_i^E) \tag{13}$$

where y_i^E is the activity of a neuron of the Gpe. Every neuron of the Gpe receives an inhibitory projection from the corresponding neuron of the NoGo part of the striatum ($w_{ii}^{EN} < 0$), and takes part to the indirect pathway. The excitation (w^{ESTN}) from the STN is part of a feedback loop to control STN activity. Due to the presence of an external input (I^E), every neuron of the Gpe is tonically active at rest.

Globus pallidus pars interna - Equations are for $i = 1, \dots, N$:

$$\tau \frac{du_i^I}{dt} = -u_i^I + w_{ii}^{IG} y_i^G + w_{ii}^{IE} y_i^E + w^{ISTN} y^{STN} + I^I \tag{14}$$

$$y_i^I = \varsigma(u_i^I) \tag{15}$$

where y_i^I is the activity of a neuron of the Gpi. Every neuron of the Gpi receives an inhibitory projection from the corresponding neuron of the Go part of the striatum ($w_{ii}^{IG} < 0$), taking part to the direct pathway, and an inhibitory projection from the Gpe ($w_{ii}^{IE} < 0$), while the excitation ($w^{ISTN} > 0$) from the STN is part of the hyperdirect way. The STN excites all the neurons of the Gpi, which in turns inhibit the corresponding neurons in the thalamus, preventing action selection.

Every neuron of the Gpi is tonically active at rest. In fact, the external input (I^I) overcomes the inhibitory input coming from the Gpe: that is the reason why the Gpi is active in the tonic state and inhibits the thalamus, although the Gpe provides inhibition to the Gpi.

Subthalamic nucleus - Since y^{STN} and u^{STN} are scalar variables, we can write :

$$\tau \frac{du^{STN}}{dt} = -u^{STN} + k^E E + \sum_{j=1}^{N} w_j^{STNE} y_j^E \tag{16}$$

with

$$E = \sum_{j=1}^{N} \sum_{\substack{i=1 \\ i \neq j}}^{N} y_i^C y_j^C \tag{17}$$

$$y^{STN} = \varsigma(u^{STN}) \tag{18}$$

where y^{STN} is the activity of the STN. The STN is connected to the cortex C and computes the conflict within it by means of an energy function E. The latter rises when two or more neurons are simultaneously active in the cortex. This is how the hyperdirect pathway starts. The projection from the Gpe is part of the feedback loop that control STN activity.

Thalamus - We have for $i = 1, \ldots, N$:

$$\tau \frac{du_i^T}{dt} = -u_i^T + w_{ii}^{TI} y_i^I + w_{ii}^{TC} y_i^C \tag{19}$$

$$y_i^T = \varsigma(u_i^T) \tag{20}$$

where y_i^T is the activity of a neuron of the thalamus. Every neuron of the thalamus receives an excitatory projection from the corresponding neuron of the cortex C ($w_{ii}^{TC} > 0$), and an inhibitory projection from the corresponding neuron of the Gpi ($w_{ii}^{TI} < 0$): the imbalance between the two projections establishes whether the corresponding action is gated or not. The excitation from the cortex to the thalamus realizes, together with the backward excitation from the thalamus to the cortex, a positive feedback loop, which is essential for cortical WTA mechanism.

Every thalamic neuron is tonically silent at rest, as a consequence of the tonic activity of the Gpi.

Cholinergic interneurons - Since y^H and u^H are scalar variables, we have:

$$\tau \frac{du^H}{dt} = -u^H + I^H + \gamma \cdot D \tag{21}$$

$$y^H = \varsigma(u^H) \tag{22}$$

where y^H is the activity of the cholinergic interneuron. The cholinergic interneuron is inhibited ($\gamma < 0$) by dopamine (D) and is tonically active at rest thanks to the input I^H.

Table S1

Parameter values of the basal ganglia (BG) model. Some parameters are related to Hebbian learning, as described by Equations (7)-(12) in the main text. : σ represents the learning factor, while ϑ^{PRE} and ϑ^{POST} are the thresholds to compute the pre-synaptic terms and the post-synaptic terms. The other parameters refer to the effect of dopamine and acethilcoline on striatal neurons, and to the neuron dynamics and sigmoid characteristics.

Name	Meaning	Value
τ / τ_L	Time constants	15[ms] / 75 [ms]
a	Parameter that sets the slope of the sigmoid	4
u_0	Central value of the sigmoid	1
ϑ^G	Threshold for dopamine activity on Go neurons	0.35
I^E	Basal external input to GPe	1
I^I	Basal external input to GPi	3
I^H	Basal external input to the cholinergic interneuron	1.0
α	Strength of the dopamine effect on Go neurons	0.75
β	Strength of dopamine effect on NoGo neurons	-1
γ	Strength of the dopamine effect on cholinergic interneuron	-0.5
σ	Learning factor in the Hebb rule	0.02
ϑ^{PRE}	Presynaptic threshold in the Hebb rule	0.5
ϑ^{POST}	Postsynaptic threshold in the Hebb rule	0.5
w_{max}	Max saturation value	0.8 (Go)<br>1.4 (NoGo)

Table S2

Synaptic values of the basal ganglia (BG) computational model before training, used to fit all patients. W^{GC}, W^{GS}, W^{NC} and W^{NS} are the synapses in the naïve conditions. These values have different values in the two-choice and four-choice programs, and have been set to maintain the value of the winner Go and NoGo neurons in the naïve state close to 0.5 before any reward or punishment (see Figure 5 in the main text).

Name	Projection	Meaning	Type	Values
$\boldsymbol{L}$	inhibition	Lateral inhibition within the cortex	extradiagonal matrix	$l_{ij} = -1.2$ ($i \neq j$)
$\boldsymbol{W^{CS}}$	excitation	From sensory cortex to motor cortex	full matrix	$w_{ii}^{CS} = 1.0$; $w_{ij}^{CS} = 1.0$ ($i \neq j$)
$\boldsymbol{W^{CT}}$	excitation	From thalamus to motor cortex	diagonal matrix	$w_{ii}^{CT} = 4$
$\boldsymbol{W^{GC}}$	excitation	From motor cortex to Go	diagonal matrix	$w_{ii}^{GC} = 0.44$ (2-choice task)<br>$= 0.4$ (4-choice task)
$\boldsymbol{W^{GS}}$	excitation	From Sensory cortex to Go	full matrix	$w_{ij}^{GS} = 0.44$ (2-choice task)<br>$= 0.4$ (4-choice task)
$\boldsymbol{W^{NC}}$	excitation	From motor cortex to NoGo	diagonal matrix	$w_{ii}^{NC} = 0.82$ (2-choice task)<br>$= 0.72$ (4-choice task)
$\boldsymbol{W^{NS}}$	excitation	From sensory cortex to NoGo	full matrix	$w_{ij}^{NS} = 0.82$ (2-choice task)<br>$= 0.72$ (4-choice task)
$\boldsymbol{W^{EN}}$	inhibition	From NoGo to GPe	diagonal matrix	$w_{ii}^{EN} = -2.2$
$\boldsymbol{W^{IE}}$	inhibition	From GPe to GPi	diagonal matrix	$w_{ii}^{IE} = -3$
$\boldsymbol{W^{IG}}$	inhibition	From Go to GPi	diagonal matrix	$w_{ii}^{IG} = -36$
$\boldsymbol{W^{TC}}$	excitation	From motor cortex to thalamus	diagonal matrix	$w_{ii}^{TC} = 3$
$\boldsymbol{W^{TI}}$	inhibition	From GPi to thalamus	diagonal matrix	$w_{ii}^{TI} = -3$
$\boldsymbol{w^{ESTN}}$	excitation	From STN to GPe	scalar	$w^{ESTN} = 1$
$\boldsymbol{w^{ISTN}}$	excitation	From STN to GPi	scalar	$w^{ISTN} = 30$
$\boldsymbol{k^{E}}$	excitation	From motor cortex to STN	scalar	$k^{E} = 7$
$\boldsymbol{W^{STNE}}$	inhibition	From GPe to STN	row vector	$w_{i}^{STNE} = -1$
$\boldsymbol{w^{GH}}$	inhibition	From cholinergic to NoGo	scalar	$w^{GH} = -1$
$\boldsymbol{w^{NH}}$	excitation	From cholinergic to Go	scalar	$w^{NH} = 1$
